# SHH Pathway Inhibition and Astrocyte Co-culture Induce Distinct Responses in Glioblastoma and Cancer Stem Cells

**DOI:** 10.21203/rs.3.rs-7214243/v1

**Published:** 2025-08-19

**Authors:** Duygu Calik Kocaturk, Berrin Ozdil, Yasemin Adali, Huseyin Aktug, Vildan Bozok, Ayşegul Uysal

**Affiliations:** Ege University; Süleyman Demirel University; Queen’s University Belfast; Ege University; Ege University; Ege University

**Keywords:** Glioblastoma (GBM), Cancer Stem Cells (CSCs), Sonic Hedgehog (SHH) Signaling Pathway, SHH Inhibition

## Abstract

Glioblastoma (GBM) represents an extremely aggressive brain malignancy with limited treatment options, difficult prognosis and a highly heterogeneous cellular architecture, including a subpopulation of cancer stem-like cells (CSCs). These CSCs frequently rely on developmental signaling pathways such as Sonic Hedgehog (SHH), which are typically dormant in adult tissue but reactivated in tumors. This study aimed to investigate how SHH pathway inhibition affects both bulk GBM cells (GBMCs) and CD133 + GBM cells (GBM CSCs), with particular emphasis on the influence of astrocyte co-culture, which more closely mimics the brain tumor microenvironment. GBMCs and GBM CSCs were cultured in mono- and astrocyte co-culture systems. They were evaluated through RT-qPCR, immunofluorescence staining, ELISA, TUNEL assay, and cell cycle analysis. By comparing treatment and culture context independently, cyclopamine-mediated SHH inhibition and astrocyte-depending signals use distinct but interacting effects on cell behavior. Cyclopamine treatment altered SHH pathway activity in a context-dependent manner, while astrocyte co-culture independently modulated GLI1, GLI3, and SUFU expression. GBM CSCs exhibited higher SHH secretion in monoculture, which was attenuated under co-culture with cyclopamine. Cell cycle analysis revealed G2/M arrest in GBMCs and G0/G1 arrest in CSCs, with astrocyte co-culture shifting CSCs toward G2/M. Apoptotic gene expression and TUNEL staining indicated enhanced extrinsic apoptosis (via CASP8) in CSCs, further intensified by SHH inhibition and co-culture. Astrocyte co-culture significantly modulates the molecular and phenotypic response of GBM cells to SHH inhibition, reshaping apoptotic and proliferative behaviors in both CSCs and bulk populations. These findings highlight the critical importance of the tumor microenvironment in therapeutic response and suggest that effective targeting of SHH signaling may require models that account for astroglial interactions.

## 1. INTRODUCTION

Glioblastoma (GBM), or grade IV astrocytoma, is the most common and aggressive brain tumor in adults, accounting for over 80% of initial malignant brain tumors^1,2^. Even with surgery, radiation, and chemotherapy, outcomes remain poor, because less than 6.8% of patients survive beyond five years^3–5^. One of the main reasons for this is the complexity of the tumor. GBM is a mosaic of many cells, including a small but highly resilient subpopulation group known as cancer stem-like cells (CSCs)^6,7^.

CSCs often activate developmental signalling pathways that are normally silent in adult brain tissue^6,8,9^. Among these, the Sonic Hedgehog (SHH) pathway can become reactivated in tumorigenic cells, supporting stemness and tumor progression^10,11^. Given the elevated SHH activity frequently observed in GBM, this pathway has emerged as a promising therapeutic target^12^. Amplified GLI1 in malignant gliomas suggests a potential link between GBM development and the SHH signaling pathway^13,14^. Cyclopamine, an alkaloid binds Smoothened (SMO)^15^, inhibits the SHH signaling pathway. Initially known for its developmental toxicity, cyclopamine has since shown anticancer potential in various models, including gliomas^16^.

Cyclopamine-mediated SHH inhibition reduces CSC viability, suppresses GBM cell colony formation in both *in vitro* cultures and *in vivo xenograft* models^13^. Cyclopamine enhances the sensitivity of CD133 + glioma stem-like cells to temozolomide and simultaneously suppresses their migratory behavior, thereby limiting their invasive potential^17^.

Co-culture systems provide a more physiologically relevant model by enabling direct cell–cell interactions and paracrine signaling, which are often absent in traditional mono-culture setups^18–20^. These interactions can influence signaling pathway activity, protein expression patterns, and cellular responses to therapeutic agents^21,22^. Importantly, cells in co-culture often exhibit altered drug sensitivity and survival dynamics, as they may support or modulate each other’s behavior through soluble factor communication, vesicle and organelle exchange^19,20,23,24^.

In this context, our primary objective was to investigate how SHH pathway inhibition affects GBM cells and GBM CSCs, both in mono-culture and in co-culture with astrocytes. To better simulate the tumor microenvironment, we employed astrocyte-containing co-culture systems and evaluated changes in SHH-related gene and protein expression, cell cycle progression, and apoptosis under cyclopamine treatment. The inclusion of astrocytes markedly influenced these responses, revealing cell type-specific shifts in proliferation and survival patterns. These findings underline the critical role of the microenvironment in shaping therapeutic outcomes and highlight the need to consider cell-cell interactions in the design of targeted strategies against GBM.

## 2. RESULTS

### 2.1 CD133 + GBM Cells Exhibit Stem-Like Morphology and Marker Expression

**Figure 1:**
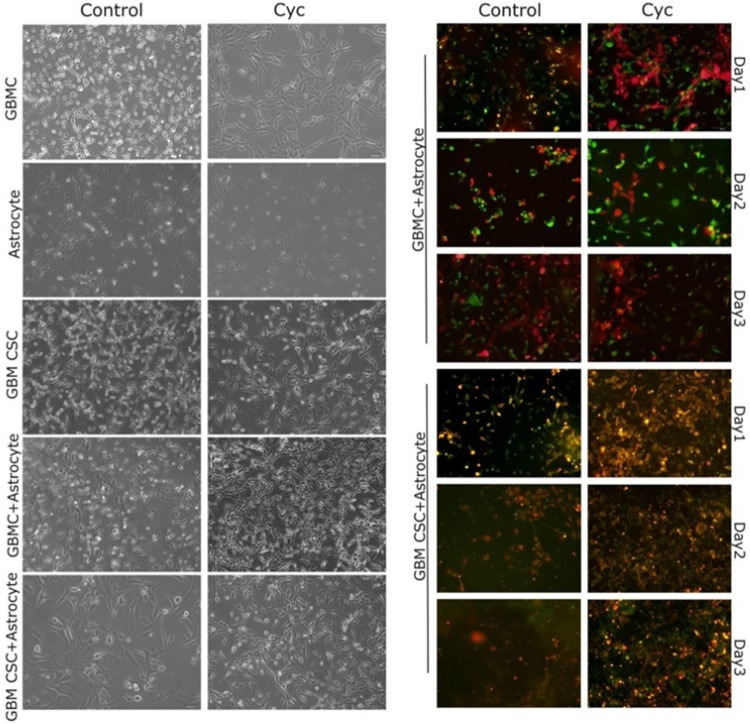
Phase-contrast and live cell imaging of GBMCs, GBM CSCs and astrocyte under cyclopamine treatment. The left panel presents representative phase-contrast images illustrating the morphological characteristics of GBMCs, GBM CSCs, and astrocytes in mono-culture, as well as GBMC–astrocyte and GBM CSC–astrocyte co-cultures, under control and cyclopamine (Cyc) conditions. The right panel displays live-cell fluorescence imaging of co-cultures labeled with CellTracker dyes. GBMCs and GBM CSCs were stained with CellTracker Green CMFDA, while astrocytes were labeled with CellTracker Orange CMRA. Images were acquired over a 3-day period (day 1–3) to monitor cell morphology and spatial organization within GBMC–astrocyte and GBM CSC–astrocyte co-culture systems, under both control and cyclopamine-treated conditions.

CD133 + cells were isolated from the U87 GBM cell line using FACS, and this subpopulation was referred to as GBM CSCs. Cells sorted for subsequent examination represented approximately 2.5% of the overall bulk population, underscoring the accurate isolation of the CD133 + cell subgroup. The expression of the stem cell-associated surface marker SSEA-1 was assessed by immunofluorescence staining and found to be consistently positive in CD133 + cells, supporting their stem-like phenotype (SI Fig. 1a). For all experiments involving the use of CSCs, we consistently selected the flow cytometry gate corresponding to the cell group stained with SSEA-1. One of the outcomes related to cell gating and sorting results is presented in SI Fig. 1b. The images of cells in mono-culture and co-culture conditions were captured using phase-contrast microscopy and are presented in Fig. 1. GBM CSCs appeared smaller in size, had a round appearance, and displayed fewer protrusions compared to GBMCs (Fig. 1). As depicted in Fig. 1, the two cell types exhibited distinct morphological characteristics; GBMCs displayed an elongated and hypertrophic appearance, whereas astrocytes demonstrated a more flattened morphology with increased surface area and spreading. Additionally, cell groups in co-culture, labeled with different fluorescent colors, were periodically imaged every 24 hours using a fluorescence microscope up to three days (Fig. 1). The cell tracker staining for mono-cultures was presented in SI Fig. 2. In both mono- and co-culture systems, the cell count increased over time. In mono-culture, clustering was notably higher, especially in the GBMCs group, while in co-culture, it was observed that GBM CSCs had more prominent interactions with astrocytes, exhibiting greater spreading on the culture surface (Fig. 1).

### 2.2 SHH Pathway and Associated Gene Expression Are Differentially Modulated by Cyclopamine in Mono- and Co-Culture Systems

**Figure 2:**
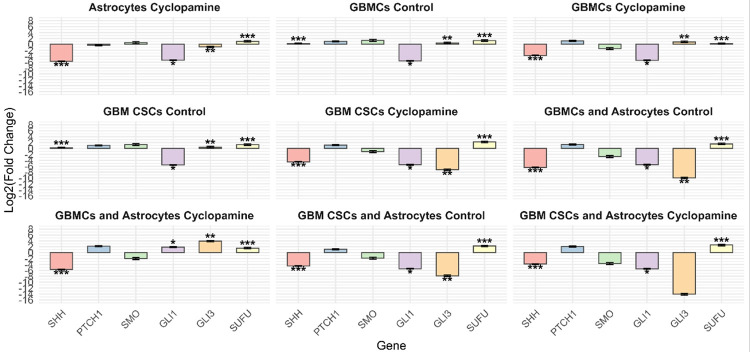
Differential expression of SHH pathway-related genes across cell types and treatment conditions. Relative mRNA expression levels of SHH, GLI1, GLI3, and SUFU were evaluated by RT-PCR in GBMCs, GBM CSCs, and their astrocyte co-cultures under control and cyclopamine-treated conditions. Gene expression values are presented as log_2_(fold change) relative to the astrocyte control group. Data are represented as mean ± SD from three biological replicates. Asterisks indicate statistically significant differences compared to the astrocyte control group (*p<0.05, **p<0.01, ***p<0.001).

Quantitative analysis of SHH pathway gene expression revealed distinct regulatory responses across different cell types and culture conditions following cyclopamine treatment, using the astrocyte control group as a baseline (Fig. 2). In astrocytes, cyclopamine induced a significant downregulation of SHH and GLI1, indicating effective inhibition of canonical pathway activation, while SUFU expression was significantly increased. In GBMCs under control conditions, SHH, GLI3 and SUFU were significantly upregulated, GLI1 was downregulated. Cyclopamine treatment maintained the suppression of SHH and GLI1, while further increase of GLI3 expression. In GBM CSCs, the control group exhibited downregulation of GLI1, alongside significant upregulation of SHH, GLI3, and SUFU. Upon cyclopamine exposure, both GLI1 and GLI3 were downregulated, while SUFU expression increased, indicating a repressor-dominant feedback regulation potentially maintaining signaling homeostasis.

Astrocyte co-culture modulated these dynamics in both cell types. In GBMC–astrocyte co-cultures, cyclopamine treatment resulted in continued downregulation of SHH, while significantly upregulating GLI3 and SUFU, suggesting that astrocyte-derived signals reinforce pathway repression while redirecting GLI-related transcriptional activity. Similarly, in GBM CSC–astrocyte co-cultures, SUFU was significantly upregulated under both control and cyclopamine-treated conditions, while SHH and GLI1 remained suppressed.

Although GBMCs and GBM CSCs showed broadly similar SHH pathway gene expression patterns in astrocyte co-cultures, cyclopamine treatment induced different regulation of GLI transcription factors. In GBM CSC–astrocyte co-cultures, both GLI1 and GLI3 were significantly downregulated following treatment. In contrast, in GBMC–astrocyte co-cultures, while GLI1 and GLI3 were downregulated under control conditions, cyclopamine exposure led to their upregulation. These findings suggest that cyclopamine triggers a distinct GLI regulatory response in GBMCs when astrocyte-derived cues are present.

**Figure 3:**
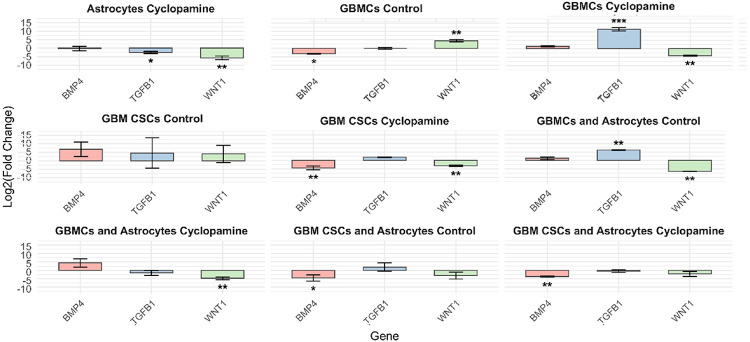
Expression profiles of WNT-related genes (WNT1, BMP4, and TGFB1) across different cell types and culture conditions relative to astrocyte controls. RT-PCR analysis was performed to assess the expression levels of WNT1, BMP4, and TGFB1 in GBMCs, GBM CSCs, and their respective astrocyte co-cultures, under control and cyclopamine-treated conditions. Gene expression values are presented as log_2_(fold change) relative to the astrocyte control group. Data represent mean±SD of three independent experiments. Asterisks indicate statistically significant changes relative to the astrocyte control group (*p<0.05, **p<0.01, ***p<0.001).

The expression of BMP4, TGFB1, and WNT1 exhibited distinct, context-dependent patterns across experimental groups, with changes evaluated relative to the astrocyte control group (Fig. 3). In astrocytes exposed to cyclopamine, both TGFB1 and WNT1 were significantly downregulated, while BMP4 remained stable. In contrast, GBMCs under control conditions showed decreased BMP4 and increased WNT1 expression. Cyclopamine treatment in GBMCs led to a notable rise in TGFB1 alongside suppression of WNT1, suggesting an inverse regulatory relationship between these pathways. This expression profile may indicate a compensatory activation of TGF-β signaling in response to WNT inhibition, potentially shaping differentiation or survival mechanisms. Interestingly, while cyclopamine consistently downregulated TGFB1 and WNT1 in astrocytes, GBMCs responded with opposing regulation of these genes, highlighting a cell type-specific transcriptional adaptation.

In GBM CSCs, all three genes-BMP4, TGFB1, and WNT1-were significantly upregulated range of expression under control conditions. However, expression levels did not differ significantly from those observed in the astrocyte control group, suggesting a baseline resemblance in differentiation-associated signaling. Upon cyclopamine treatment, both BMP4 and WNT1 were markedly downregulated, indicating partial suppression of differentiation and WNT-related pathways in CSCs. These results suggest that, unlike GBMCs, CSCs exhibit a more uniform downregulation pattern upon SHH pathway inhibition, which may reflect their intrinsic reliance on WNT and BMP signaling for maintaining stem-like features.

Astrocyte co-culture further modulated these transcriptional responses in a cell type-specific manner. In GBMC–astrocyte co-cultures, *TGFB1* was upregulated and *WNT1* was downregulated under control conditions; cyclopamine exposure further suppressed *WNT1* expression. In contrast, GBM CSC-astrocyte co-cultures showed downregulation of *BMP4* even under control conditions, with cyclopamine inducing an even more pronounced decrease. Together, these findings underscore that astrocyte-derived cues shape the balance between pro-differentiation and self-renewal pathways differently in GBMCs and GBM CSCs, especially when pharmacological inhibition is introduced.

**Figure 4:**
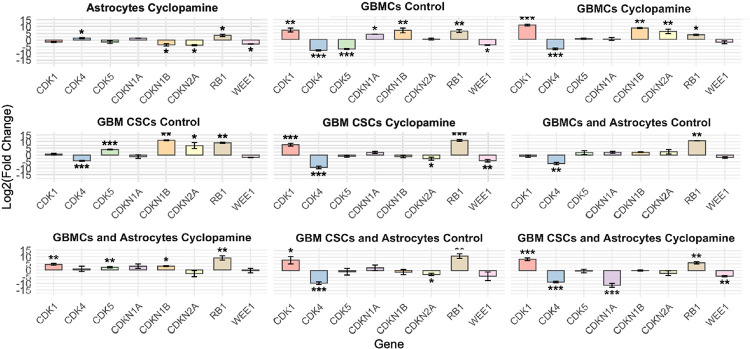
Expression profiles of cell cycle-related genes across different cell types and treatment conditions relative to astrocyte controls. RT-PCR analysis was performed to evaluate the expression of key regulators of cell cycle progression (CDK1, CDK4, CDK5, CDKN1A [p21], CDKN1B [p27], CDKN2A [p16], RB1, and WEE1) in GBMCs, GBM CSCs, and their astrocyte co-cultures under control and cyclopamine-treated conditions. Gene expression values are shown as log_2_(fold change) relative to the astrocyte control group. Data represent mean±SD of three independent experiments. Asterisks denote statistically significant differences compared to astrocyte control (*p<0.05, **p<0.01, ***p<0.001).

Cell cycle-associated genes showed distinct transcriptional responses to cyclopamine across cell types and culture conditions, with fold changes calculated relative to the astrocyte control group (Fig. 4). In astrocytes, cyclopamine treatment led to significant upregulation of CDK4 and RB1, while CDKN1B, CDKN2A, and WEE1 were significantly downregulated, indicating potential modulation of G1/S transition regulators.

In GBMCs under control conditions, CDK1, CDKN1A, CDKN1B, and RB1 were significantly upregulated, whereas CDK4, CDK5 and WEE1 were downregulated. This profile suggests enhanced cell cycle progression coupled with checkpoint activation. Upon cyclopamine treatment, CDK1, CDKN1B, CDKN2A, and RB1 remained significantly elevated, suggesting alternative regulatory pathways that maintain cell cycle progression despite loss of canonical SHH input. Between control and cyclopamine-treated GBMCs, CDK5 and WEE1 expression returned to baseline levels, while CDKN2A expression was upregulated.

In GBM CSCs, cell cycle gene expression revealed a distinct regulatory profile relative to astrocyte controls. Under control conditions, CDK4 was significantly downregulated, while CDK5, CDKN1B, CDKN2A, and RB1 were upregulated, suggesting checkpoint activation and potential entry into a quiescent state. In GBM CSCs, cyclopamine treatment induced significant changes in the expression of genes involved in cell cycle regulation. CDK1 and RB1 were significantly upregulated, while CDK4, CDKN2A, and WEE1 were markedly downregulated. The increase in CDK1 and RB1, combined with suppression of WEE1, suggests that cells may be transcriptionally primed for mitotic entry. However, the concurrent reduction in CDK4 and CDKN2A indicates a complex regulatory landscape, possibly involving both proliferative and inhibitory signals. These findings, together with cell cycle phase data showing G0/G1 accumulation (below in cell cycle result), point to a partial disconnection between mitotic gene induction and actual cell cycle progression, likely reflecting checkpoint adaptation or stress-related transcriptional compensation in response to SHH pathway inhibition.

Astrocyte co-culture further influenced these dynamics. In GBMC and astrocyte co-cultures, control situation shows CDK4 downregulation and RB1 upregulation. Cyclopamine induced the upregulation of CDK1, CDK5, CDKN1B, and RB1. This may reflect a coordinated activation of G1/S and G2/M checkpoints in response to microenvironmental cues. In GBM CSC and astrocyte co-cultures, CDK1 and RB1 were also significantly upregulated following treatment, while CDK4 and CDKN2B, were significantly suppressed. These findings indicate that astrocyte-derived signals modify the transcriptional response to SHH inhibition in a cell type–specific manner, reinforcing or redirecting cell cycle control programs in GBM subpopulations. When comparing GBMCs in monoculture and co-culture with astrocytes, the expression levels of CDK1, CDK5, CDKN1A, CDKN1B, and WEE1 in the co-culture condition were similar to those observed in the astrocyte control group, suggesting a normalizing influence of astrocyte-derived signals on cell cycle gene regulation. In GBM CSCs, CDK1 expression was elevated under co-culture conditions, whereas the expression of other cell cycle-related genes-excluding CDK4 and RB1-remained comparable to astrocyte controls. Notably, CDK4 consistently exhibited downregulation, and RB1 remained upregulated across both monoculture and co-culture conditions, suggesting that these regulatory patterns are preserved independently of astrocyte-derived cues.

**Figure 5:**
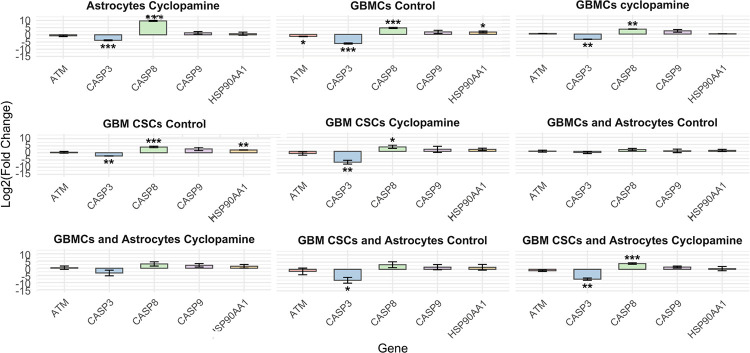
Expression profiles of apoptosis-related genes across different cell types and treatment conditions relative to astrocyte controls. Data represent mean ± SD of three independent experiments. Asterisks indicate statistically significant changes compared to the astrocyte control group (*p<0.05, **p<0.01, ***p<0.001).

Quantitative RT-PCR analysis of apoptotic genes (ATM, CASP3, CASP8, CASP9, and HSP90AA1), was performed in GBMCs, GBM CSCs, and their co-cultures with astrocytes under control and cyclopamine-treated conditions. Gene expression levels are presented as log_2_(fold change) relative to the astrocyte control group. Apoptosis-associated genes displayed differential expression patterns across cell types and culture conditions following cyclopamine treatment, evaluated relative to the astrocyte control group (Fig. 5). In astrocytes, cyclopamine significantly downregulated CASP3 while strongly upregulating CASP8, suggesting selective activation of the extrinsic apoptotic pathway. In GBMCs, control conditions led to increased expression of CASP8 and HSP90AA1, while ATM and CASP3 were markedly downregulated. Cyclopamine treatment further suppressed CASP3 and upregulated CASP8, indicating sustained activation of death receptor signaling under SHH inhibition. In GBM CSCs, control cultures showed upregulation of CASP8 and HSP90AA1, with significant downregulation of CASP3. Cyclopamine further decreased CASP3 expression while maintaining CASP8 activation, again implicating extrinsic apoptotic responses.

Astrocyte co-cultures modified these dynamics in a cell-type-dependent manner. In GBMC and astrocyte co-cultures, no statistically significant changes were observed for the apoptosis-related genes. However, in GBM CSC and astrocyte co-cultures, CASP3 was significantly downregulated under control conditions and remained suppressed following cyclopamine exposure, while CASP8 was strongly upregulated. These observations collectively indicate that cyclopamine predominantly activates the extrinsic apoptosis pathway via CASP8, especially in GBM CSC populations, suggesting that apoptosis is differentially regulated depending on cellular and environmental context. Although GBMCs and GBM CSCs exhibited distinct CASP3 and CASP8 expression profiles under mono-culture conditions, these differences appeared to moderate when the cells were co-cultured with astrocytes. In this combined environment, both cell types displayed expression patterns more closely resembling those of astrocyte controls, suggesting that astrocyte-derived cues may play a stabilizing role in the regulation of apoptotic gene expression and attenuate cell-type-specific variability.

### 2.3 SHH Pathway Proteins Are Differentially Expressed in GBMCs and GBM CSCs

**Figure 6:**
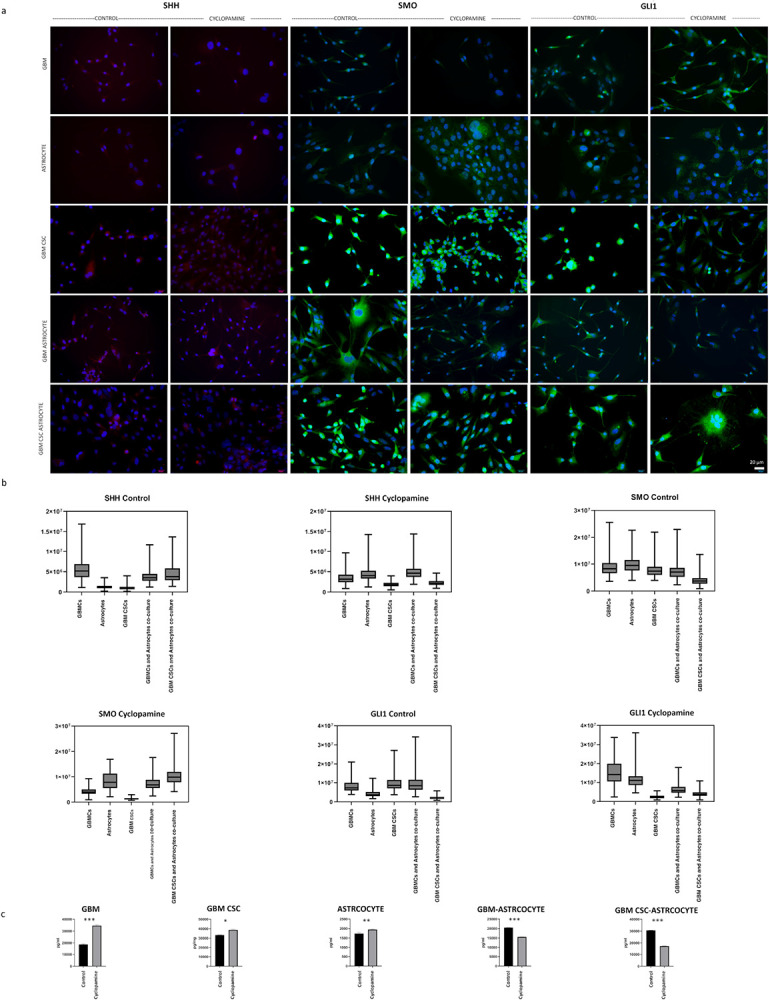
Protein expression level comparison a) Immunofluorescence images of SHH, SMO and GLI1 protein b) their fluorescence intensity comparison and c) SHH secretion. The intensity values of SHH and GLI1 proteins varied with cyclopamine treatment. For SMO protein, the intensity values changed in all groups except for the GBMCs and astrocyte co-culture. Notably, in the GBM CSCs group, it was demonstrated that the response of the astrocyte co-culture to cyclopamine treatment altered the intensity values of the proteins. Statistical significance was considered at*p < 0.05, **p < 0.01, ***p < 0.001.

Here, the cyclopamine was used to investigate the expression of SHH, SMO, and GLI1 proteins in both mono- and co-cultured GBM and astrocytes within the SHH signaling pathway (Fig. 6). A Wilcoxon signed-rank test showed that there is no sufficient evidence to say that GBMCs and astrocytes co-culture is statistically significant difference between control and cyclopamine groups in SMO immunofluorescence intensity with cyclopamine (Z= −0.217, p = 0.83) (SI Table 1). However, the difference between cyclopamine and control groups was statistically significant for all other experimental cell groups and immunofluorescence intensity for SMO, SHH and GLI (SI Table 1). Additionally, Kruskal- Wallis H test showed that there is statistically significant difference in the median SMO, SHH and GLI1 immunofluorescence intensity between cell culture experimental groups, if there is a cyclopamine treatment (Respectively; X^2^(4) = 492, X^2^(4) = 391, X^2^(4) = 497, p = 0.001) (SI Table 2). In addition, same analysis shows that there is statistically significant difference in the median SMO, SHH and GLI1 immunofluorescence intensity between experimental groups, if there is no cyclopamine treatment (Respectively; X^2^(4) = 346, X^2^(4) = 413, X^2^(4) = 405, p = 0.001) (SI Table 2).

### 2.4 SHH Secretion Is Elevated in GBM CSCs and Suppressed by Cyclopamine

The ELISA test was conducted utilizing the cell culture supernatants. The objective was to detect changes in SHH paracrine secretion between groups relying on the cell culture supernatants (Fig. 6c). According to the ELISA results, the presence of SHH in supernatants varied across groups. The lowest SHH protein levels were observed in the astrocyte control group, while the highest levels were detected in the GBM CSCs cyclopamine group. Among the control groups, the highest SHH protein presence in the supernatants was in the GBM CSCs control group. In terms of paracrine SHH protein content within the culture supernatants, the control groups can be ordered from the highest to the lowest as follows: GBM CSCs control group (33406 pg/ml), GBM CSCs and astrocytes co-culture group (30660 pg/ml), GBMCs and astrocytes co-culture control group (20513 pg/ml), GBMCs control group (18720 pg/ml), and astrocytes control group (1737 pg/ml). Regarding the cyclopamine-treated groups, the order of SHH presence in the supernatants, from highest to lowest, was as follows: GBM CSCs cyclopamine group (38723 pg/ml), GBMCs cyclopamine group (34611 pg/ml), astrocytes (1946 pg/ml), GBM CSCs and astrocytes co-culture group (17350 pg/ml) and GBMCs and astrocytes co-culture group (15536 pg/ml). In summary, the secretion pattern indicates that there is a higher presence of SHH protein in GBMCs and GBM CSCs compared to the astrocytes in the supernatants. In addition, while mono-cultures increased the secretion of SHH after cyclopamine treatment; co-cultures decreased the secretion of SHH protein.

### 2.5 Cyclopamine Induces G_0_/G_1_ Arrest in GBM CSCs

**Figure 7:**
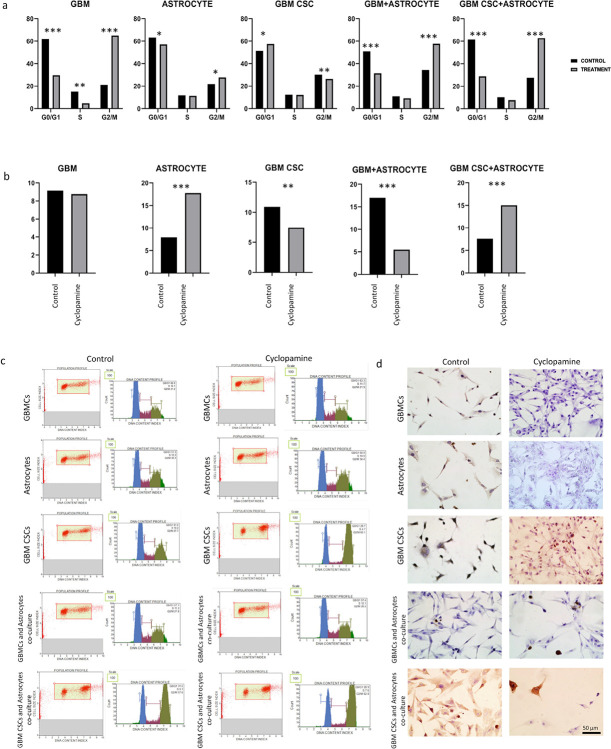
Assays for cellular processes as cell cycle (a and c), and TUNEL (b and d). The TUNEL images of the cell groups. Statistically significant difference was defined as *p < 0.05, **p < 0.01, ***p < 0.001.

Cell cycle analysis has clearly demonstrated the responses of cell groups to treatment (Fig. 7a, c). When compared as monocultures, GBMCs and astrocytes exhibited an increase in the G2/M phase upon cyclopamine treatment, whereas GBM CSCs showed a tendency towards the G0/G1 phase. According to co-culture analyses, the GBMC and astrocyte co-culture group responded to cyclopamine with G2/M arrest. Interestingly, while GBM CSCs in monoculture did not show a change due to cyclopamine treatment, they accumulated in the G0/G1 phase. However, in the GBM CSC and astrocyte co-culture, cyclopamine induced G2/M phase arrest. This clearly indicates that co-culture alters the drug response and resistance dynamics in both the bulk population and the co-culture set-up.

### 2.6 SHH Inhibition Enhances Apoptosis in GBM CSCs

The aim was to identify and stain DNA fragmentation occurring during apoptotic cell death using the TUNEL method. In the context of SHH inhibition, this staining was employed to assess changes in apoptotic levels of cells in both SHH inhibition and co-culture conditions (Fig. 7b, d). In both the GBMCs and GBM CSCs control groups, the rate of TUNEL-positive cells was notably higher compared to the groups treated with cyclopamine (9.15% vs 8.76; 10.92% vs 7.45, respectively), indicating a reduction in apoptotic activity in response to the cyclopamine. Conversely, in the astrocytes control group, a significant rise in the proportion of TUNEL-positive cells was observed following drug treatment (7.92% vs 17.76%), exhibiting a distinct pattern from that of GBMCs and GBM CSCs. Furthermore, in the context of the GBMCs and astrocyte co-culture control group, a decline in the count of TUNEL-positive cells was evident in the drug-treated subset (17% vs 5.49%). Conversely, in the GBM CSCs and astrocyte cell co-culture group, there was an observable increase in the number of TUNEL-positive cells in the drug-treated group when compared to the control group. These findings demonstrates the differential apoptotic responses of these cell populations to cyclopamine treatment. In the TUNEL assay results, it was determined that GBMCs are resistant to cyclopamine compared to GBMCs with astrocytes and they resist apoptosis. Additionally, it was observed that the presence of astrocytes results in different responses to cyclopamine, with GBMCs increasing while GBM CSCSs preferring the apoptotic pathway.

## 3 DISCUSSION

GBM continues to represent one of the most challenging malignancies in neuro-oncology, not only because of its aggressive clinical course, but also due to the remarkable cellular diversity within the tumor, including therapy-resistant CSCs^28^. Over the past decade, developmental signaling pathways such as SHH, which are normally quiescent in adult brain tissue, have been increasingly recognized for their reactivation and oncogenic contributions in GBM^12,29^. Dysregulation of SHH components, such as SHH ligands and downstream effectors like GLI transcription factors, has been associated with increased proliferation, invasion, and resistance to treatment in cancer^13,30^. While much is known about SHH signaling in isolated cancer cells, less is understood about how these pathways operate within the context of the tumor microenvironment-particularly in the presence of astrocytes, which are abundant in the brain and interact closely with tumor cells. Previous studies have shown that the SHH pathway, particularly GLI1, influences tumorigenesis in both cancer cells and cancer stem cells^31^. In this study, we took an integrated approach to explore how SHH pathway inhibition with cyclopamine reshapes molecular and cellular behavior in both GBMCs and GBM CSCs. By combining gene and protein expression profiling with apoptosis and cell cycle analyses across monoculture and astrocyte co-culture systems, we aimed to uncover not just the direct effects of pathway inhibition, but also how microenvironmental signals influence these therapeutic responses.

The differential outcomes observed in co-cultured cells compared to monocultures suggest that cells within co-culture systems are more amenable to *in vivo*-like mimicry. This highlights the therapeutic significance of advancing these systems. In Schmitt et. al study, primary cells were established, and co-culture experiments were conducted. Specifically, primary GBMCs were subjected to co-cultivation alongside astrocytes and microglial cells, and the levels of chemokines and cytokines were examined. The outcomes derived from this comprehensive study revealed notable differences when contrasted with conventional mono-culture approaches^32^. This study represents one of the investigations that elucidate the molecular-level differences in cell-to-cell communication observed in co-culture experiments, which has implications for pre-animal experimental research. A study based on mitochondrial research has established that GBM cells induce their aggressive character through horizontal mitochondrial transfer from surrounding cells^24^. This indicates that the co-culture is influenced not only by the presence of other cell types besides GBM but also by the intracellular content of these cells.

Building upon our findings, our RT-PCR analyses revealed context-specific transcriptional responses to SHH pathway inhibition across GBMCs and GBM CSCs under both mono-culture and astrocyte co-culture conditions. GLI3, which has been previously shown to be highly expressed in cancer stem-like populations^33,34^, was especially responsive in co-culture, reinforcing the regulatory influence of astrocyte-derived cues. At the protein level, immunofluorescence confirmed decreased SHH and GLI1 intensities following cyclopamine exposure. This finding supports earlier observations in GBMCs, where cyclopamine inhibited GLI1 nuclear translocation and prevented spheroid formation^17^. Additionally, astrocyte co-culture altered SHH protein intensity in GBM CSCs, suggesting that microenvironmental factors modulate protein-level responses. The differential response of glioma stem cells to SHH ligand, as compared to non-stem glioblastoma cells, highlights the potential of SHH pathway-targeted approaches as an effective single-cell killing strategy specifically against the stem-like tumor subpopulation^31^. Here, ELISA measurements indicated high SHH secretion by GBM CSCs, which may account for lower intracellular SHH levels observed in GBMCs. Notably, co-culture with astrocytes reduced SHH secretion in both cell types, while astrocytes themselves maintained low SHH expression, in line with prior reports.

Analysis of BMP/TGFβ/WNT-related transcripts revealed distinct patterns depending on cell type and culture condition. In GBMCs co-culture with astrocytes led to WNT1 downregulation and TGFB1 upregulation, while WNT1 upregulation when compared to mono-culture. In contrast, in GBM CSC and astrocyte co-cultures, BMP4 decreased. These data emphasize the context-specific modulation of developmental pathways by both CSC characteristics and astrocytic signals. BMP4’s known role in promoting differentiation and anti-proliferative effects in GBM CSCs^35,36^ and its reciprocal inhibition with TGFβ1 in other cancer types may explain this inverse regulation^37^. Moreover, the critical role of WNT and BMP4 in neural tumors such as neuroblastoma^38^ and their modulation by SHH inhibitors further supports our findings. The observed elevation of TGFB1 expression in GBMCs, especially under monoculture, is also in agreement with prior transcriptomic analyses^39^. Our results suggest that astrocyte interactions can either buffer or strengthen pathway-specific transcriptional programs, possibly redirecting cell fate decisions.

Apoptosis-related gene expression data revealed that CASP8 was strongly upregulated in GBM CSCs, suggesting activation of extrinsic apoptosis pathways supported by TUNEL assay. However, in co-culture settings, expression of both CASP3 and CASP8 normalized toward astrocyte control levels, while TUNEL positivity declined, indicating a protective influence of astrocytic microenvironmental signals. These findings align with the concept that astrocytes modulate cell death mechanisms and buffer stress responses, particularly in CSCs.

Cell cycle regulation also reflected complex transcriptional and functional interplay. SHH signaling has previously been linked to the maintenance of G1 cyclin expression and continuous cycle progression^40^. In our model, cyclopamine-treated GBMCs exhibited CDK1 and CDKN2A upregulation, along with increased G2/M phase occupancy. In GBM CSCs, however, CDK1 and RB1 were upregulated while CDK4 and WEE1 were suppressed, yet the G0/G1 population increased, suggesting checkpoint activation. Interestingly, co-culture restored several gene expression levels (CDK1, CDK5, CDKN1A) to baseline, while CDK4 remained suppressed and RB1 remained elevated, implying the presence of core regulatory mechanisms unaffected by external signals. These findings indicate that astrocyte-derived cues modulate the magnitude and direction of gene expression in GBM cells, but certain intrinsic features, especially those regulating G1/S transition and quiescence, persist independently.

Taken together, our gene and protein expression results—integrated with TUNEL and cell cycle data—demonstrate that the presence of astrocytes substantially alters the transcriptional and phenotypic response to SHH inhibition in both GBMCs and GBM CSCs. This highlights the importance of modeling tumor–stroma interactions to improve the translational relevance of therapeutic targeting studies in GBM.

## 4 CONCLUSION

By introducing astrocyte co-culture, this study demonstrates that SHH pathway inhibition with cyclopamine stimulates markedly different responses in GBM and their sub-populations, offering new insight into how microenvironmental cues shape treatment outcomes. The co-culture environment substantially reshaped gene expression dynamics, protein localization, and functional outcomes such as cell cycle distribution and apoptosis, underscoring the critical influence of the tumor microenvironment. Notably, astrocyte-derived cues altered GLI1 and GLI3 responses, in GBMC–astrocyte co-cultures, cyclopamine reversed downregulation to upregulation, indicating a distinct GLI regulatory adaptation and modulated SHH secretion, and influenced apoptotic sensitivity in a cell-type-specific manner. Co-culture also modulated differentiation-related pathways, with downregulation of WNT1 and selective upregulation of TGFB1, suggesting that astrocytes influence GBMCs fate decisions through pathway-specific transcriptional shifts. In terms of cell cycle control, astrocyte co-culture led to basal level except CDK4 and RB1 enhancement in GBMCs, implying checkpoint reinforcement. Importantly, our findings suggest that GBM CSCs remain highly responsive to extrinsic apoptotic signaling under SHH inhibition, especially when microenvironmental signals from astrocytes are present. Furthermore, the distinct shifts in cell cycle progression, particularly the G0/G1 accumulation in GBM CSCs and G2/M arrest in GBMCs, point to differential checkpoint engagement, potentially reflecting adaptive resistance mechanisms. Together, these results emphasize the importance of modeling tumor–stroma interactions in preclinical research and highlight the limitations of monoculture systems in capturing the complexity of GBM biology. Targeting developmental pathways such as SHH in a context-aware manner, accounting for astrocyte-mediated modulation, while accounting for the separate contributions of microenvironmental factors like astrocytes, may improve therapeutic precision against glioblastoma and its stem-like subpopulations. As a future perspective, investigation of astrocyte organelle dynamics, particularly mitochondria, may reveal novel mechanisms regulating GBM cell sensitivity and resistance within the microenvironment.

## 5 MATERIALS AND METHODS

### 5.1 Cells Lines and Culture Conditions

Human astrocytes and GBM cell lines were represented with SVG p12 (ATCC CRL-8621) and U87 MG (ATCC HTB-14), respectively. Cells were cultured in Eagle’s Minimum Essential Medium (EMEM; Sigma M4655), supplemented with 10% fetal bovine serum (FBS; Gibco 10270–106) and 1% penicillin-streptomycin (Thermo 15140122). Cultures were maintained at 37°C in humidified incubator with 5% CO_2_. Cells were passaged at 80% confluency using trypsin-EDTA (Sigma SLB56658). Passage numbers 4 to 6 were used for all experiments. Cell viability was assessed using the ‘Muse Cell Count & Viability Kit’ (Millipore MCH600103).

### 5.2 Isolation of CD133 + GBMC via Fluorescence-activated Cell Sorting (FACS)

GBM CSCs were isolated from U87 MG cells based on CD133 surface expression using FACS as previously described^25^. Cells were incubated with CD133-phycoerythrin (PE)-conjugated antibody (Miltenyi Biotec, 130-113-186) and DAPI for 12 minutes at 4°C in the dark. After incubation, cells were washed with phosphate-buffered saline (PBS) supplemented with 1% dialyzed FBS (Biowest, S1810). Sorting was performed using a FACSAria II flow cytometer (BD Biosciences). Cells from passages up to three were used for sorting.

Following isolation, CD133 + cells were referred to as GBM CSCs in subsequent experiments. The stem-like phenotype of the sorted population was further validated by immunostaining for stage-specific embryonic antigen-1 (SSEA-1) (see SI Fig. 1).

### 5.3 Co-Culture Design

In mono-culture experiments, GBM cells, GBM CSCs, or astrocytes were seeded individually at a density of 1×10^4^ cells/mL. For co-culture setups, either GBMCs or GBM CSCs were combined with astrocytes at equal proportions (5×10^3^ cells/mL per cell type), resulting in a total seeding density of 1×10^4^ cells/mL. The following co-culture combinations were established: (i) GBMCs with astrocytes and (ii) GBM CSCs with astrocytes.

Cells were gently mixed prior to plating to ensure uniform distribution and seeded in standard tissue culture plates. All co-cultures were maintained under the same medium and incubation conditions as the mono-cultures. Cell viability and cell–cell interactions were monitored throughout the culture period to confirm stable co-culture dynamics.

To determine the effective inhibitory concentration of cyclopamine on SHH signaling, GBMCs and GBM CSCs were treated with a range of doses (100, 50, 10, 5, and 1 μM) and assessed at 24, 48, and 72 hours using the MTT assay (Thermo Fisher Scientific, V13154). The IC_50_ concentration was identified as 5 μM. For subsequent experiments, for all cell groups, cyclopamine (Selleckchem, S1146) was administered at 5 μM, 24 hours after cell seeding. The compound was directly added to the culture medium and remained present throughout the incubation period to ensure sustained SHH pathway inhibition.

### 5.4 Live Cell Staining

To visualize cell distribution and interaction dynamics in co-culture settings, live-cell imaging was performed over a 72 hours period. Cells were seeded at a density of 5×10^5^ cells/mL per group in 6-well plates and labeled using CellTracker dyes. Astrocytes were stained with CellTracker Orange CMRA dye (Thermo Fisher, C34551), while GBM cells and GBM CSCs were stained with CellTracker Green CMFDA dye (Thermo Fisher, C7025). Prior to staining, cells were incubated for 30 minutes in serum-free medium containing the respective dyes, in the dark. After staining, culture medium containing 10% FBS was added, and cells were incubated under standard conditions (37°C, 5% CO_2_). Fluorescence images were acquired using an inverted fluorescence microscope at 24 hours intervals, ensuring consistent observation across all groups.

### 5.5 RNA Isolation and Quantitative RT-PCR

Total RNA was extracted using the High Pure RNA Isolation Kit (Roche, 11828665001), following the manufacturer’s instructions. RNA purity and concentration were assessed spectrophotometrically using a NanoDrop device, and only samples with A260/280 ratios between 1.8 and 2.0 were used. Complementary DNA (cDNA) was synthesized using the EvoScript Universal cDNA Master kit (Roche, 7912374001). Gene expression analysis was performed using a RealTime ready Custom Panel (Roche, 5582571001) designed to assess genes related to the SHH pathway, cell cycle regulation, apoptosis, and differentiation. The FastStart Essential DNA Probes Master (Roche, 6402682001) was used for amplification reactions. Quantitative PCR was carried out in accordance with the kit protocol. GAPDH served as the endogenous control for normalization. Target genes included SHH, PTCH1, SMO, SUFU, GLI1, and GLI3 (SHH pathway); WNT1, BMP4, and TGFB1 (differentiation); CDK1, CDK4, CDK8, RB1, CDKN1A, CDKN2A, and WEE1 (cell cycle); and ATM, NFKB1, CASP3, CASP8, CASP9, and HSP90AA1 (apoptosis). Primer sequences for each gene are listed in Table 1.

Relative expression levels were calculated using the 2^ΔΔCt^ method, with the astrocyte mono-culture group used as the reference condition. All quantitative RT-PCR reactions were performed in triplicate. Statistical comparisons between groups were conducted using Student’s t-test, and p-values less than 0.05 were considered statistically significant.

### 5.6 Immunofluorescent Staining

Immunofluorescence staining was performed to assess the expression of SHH pathway components (SHH, SMO, and GLI1) and stem cell (SSEA-1) marker following by the previous procedure^26^. Cells were fixed with 4% paraformaldehyde at 4°C, followed by permeabilization with 0.25% Triton X-100 for 15 minutes. Non-specific binding was blocked by incubating samples with 1% bovine serum albumin (BSA) for 1 hour at room temperature. Primary antibodies were diluted 1:100 in 1% BSA in 1X PBS and incubated overnight at 4°C. The following antibodies were used: anti-SSEA-1 (Santa Cruz, sc-21702), anti-SHH (Santa Cruz, sc-373779), anti-GLI1 (Bioss, AI05040), and anti-SMO (Bioss, AA062588). After washing, cells were incubated with fluorophore-conjugated secondary antibodies (1:200; Anti-mouse, Thermo Fisher A-21422; Anti-rabbit, Thermo Fisher A-11034) for 1 hour at room temperature. Nuclei were counterstained using Fluoroshield Mounting Medium with DAPI (Abcam, ab104139). Fluorescence images were captured using an Olympus BX-51 microscope (Olympus Optical Co., Tokyo, Japan). For each group, at least three biological replicates were analyzed, and fluorescence intensity was quantified from a minimum of 100 cells across 10 fields per replicate with ImageJ/Fiji program. Statistical comparisons were performed using the Kruskal–Wallis test and Wilcoxon signed-rank test, with significance defined as p < 0.05. Detailed results are provided in SI Tables 1–2 and visualized in Fig. 6.

### 5.7 ELISA Assay for Sonic Hedgehog Secretion

ELISA analysis was performed using a commercially available human SHH ELISA kit (Cusabio CSB-E12005h) to investigate the paracrine release of SHH. Culture supernatants were collected after 72 hours of incubation from both mono- and co-culture conditions and treated and non-treated conditions. Prior the assay, all reagents were equilibrated to room temperature (Cusabio CSB-E12005h). Subsequently, 100 μL of both standards and samples were added to their respective wells and incubated at room temperature for 2.5 hours with gentle shaking. After this incubation period, the solution in the wells was discarded, and the samples were washed with a washing solution. Following the wash, 100 μL of the ‘SHH Detection’ antibody was added to each well and incubated for 1 hour at room temperature with gentle shaking. Another washing step was performed after this incubation. After the final wash, 100 μL of HRP-Streptavidin solution was added to each well and incubated for 45 minutes at room temperature with gentle shaking. Following another washing step, 100 μL of 3.3’, 5.5’-tetramethylbenzidine (TMB) single-step substrate reagent was added to each well. This mixture was then incubated for 30 minutes at room temperature with gentle shaking in the dark. Finally, 50 μL of the termination solution was added to each well, and the samples were read at 450 nanometers by Multiskan Fc Microplate Photometer (Thermo Scientific). All measurements were performed in duplicate, and results are presented as mean ± standard deviation.

### 5.8 Cell Cycle Analysis

Cell cycle distribution was assessed using the Muse Cell Cycle Kit (Millipore, MCH100106), following the manufacturer’s protocol. The shift in the percentage distribution across cell cycle phases, both in the presence and absence of cyclopamine, was thoroughly investigated. After 72 hours of incubation under respective treatment conditions, cells were harvested, washed with PBS, and fixed in 70% ethanol at −20°C. Fixed cells were incubated with the cell cycle reagent containing propidium iodide and RNase A (Millipore MCH100106) for 30 minutes, then analyzed using the Muse Cell Analyzer (Millipore). The percentage of cells in G0/G1, S, and G2/M phases were determined based on DNA content. Each condition was analyzed in triplicate.

### 5.9 Terminal deoxynucleotidyl transferase dUTP Nick End Labeling (TUNEL) Assay

TUNEL assay was performed using the In Situ Cell Death Detection Kit, Fluorescein (Roche, 11684795910) to detect DNA fragmentation as a marker of apoptosis. Cells were fixed with 4% paraformaldehyde and treated with 3% H_2_O_2_ in methanol at room temperature for a 10 minutes duration to block endogenous peroxidase activity. Subsequently, 0.1% Triton-X100 solution was applied to the cells for 2 minutes on ice to facilitate permeabilization. Following permeabilization, in accordance with the TUNEL staining kit protocol (Roche, 11684817910), 9 units of Label solution and 1 unit of Enzyme solution were mixed to create the TUNEL reaction mixture. This TUNEL reaction mixture was then applied to the samples for 1 hour at 37°C. Following the TUNEL reaction, “Converter-POD” was applied to the samples for 30 minutes at 37°C. For staining, all samples were treated with DAB (3,3’-Diaminobenzidine) and subsequently with hematoxylin. Imaging was carried out using the Olympus C-5050 Digital Camera BX51 Microscope. To analyze the TUNEL assay data, a minimum of 100 cells from 10 images obtained from three independent setup were counted in each group. Cells exhibiting a staining pattern indicative of apoptosis in each area were considered positive for apoptotic index, and the percentage ratio was determined by positive to total number of cell ratio. In the TUNEL staining analysis, cells were categorized as positive if they exhibited a staining pattern indicative of apoptosis within each specified area. The evaluation was performed by three separate histologists. Comparisons of the groups were subsequently conducted based on this apoptosis criterion^27^. Results were expressed as the percentage of TUNEL-positive cells, and statistical significance was determined using the Kruskal–Wallis test (p < 0.05).

### 5.10 Statistical Analysis

Gene expression data were analyzed using the 2^–ΔΔCT^ method, where ΔΔCT was determined as [(CT of the gene – CT of GAPDH)_for the treated group_] – [(CT of the gene – CT of GAPDH)_for the control group_]. GAPDH was used as the internal reference gene for normalization during the analysis. The comparisons were statistically performed using Student’s t-test. The expression levels were quantified through fold change and fold regulation. In this analysis, the astrocyte control group was compared to other experimental groups. Fold change was calculated as (Fold change= (Expression level of the experimental group)/(Expression level of the control group)), with the astrocyte control group serving as the baseline control. Fold regulation was determined using the formula: Fold Regulation = −1/(Fold change).

STATA IC 16.1 (StataCorp, 4905 Lakeway Drive, College Station, Texas 77845 USA) was used for all statistical analyses for immunofluorescence staining, ELISA, cell cycle and TUNEL assay. The Shapiro-Wilk test of the normality of the distribution of the measured parameters served as the first step in the statistical analysis. Due to the non-normality of the immunofluorescence intensity variables, Kruskal-Wallis analysis of variance and Wilcoxon signed rank tests were used to identify dependencies. The graphs were created using GraphPad Prism version 9.3.0 software and R Studio version 4.4.1.

## Supplementary Material

Supplementary Files

This is a list of supplementary files associated with this preprint. Click to download.

- SITablesandfigures.docx

## Figures and Tables

**Table 1:** List of forward and reverse primers used for the amplification of genes involved in SHH signaling, cell cycle regulation, differentiation, and apoptosis.

Genes	Forward	Reverse
SHH	CCGAGCGATTTAAGGAACTCACC	AGCGTTCAACTTGTCCTTACACC
PTCH1	GCTGCACTACTT CAGAGACT GG	CACCAGGAGTTTGTAGGCAAGG
SMO	TGCTCATCGTGGGAGGCTACTT	ATCTTGCTGGCAGCCTTCTCAC
SUFU	CAGCAAACCTGTCCTTCCACCA	CAGATGTACGCTCTCAAGCTGC
GLI1	AGCCTTCAGCAATGCCAGTGAC	GTCAGGACCATGCACTGTCTTG
GLI3	TCAGCAAGTGGCTCCTATGGTC	GCTCTGTTGTCGGCTTAGGATC
WNT1	CTCTTCGGCAAGATCGTCAACC	CGATGGAACCTTCTGAGCAGGA
BMP4	CTGGTCTTGAGTATCCTGAGCG	TCACCTCGTTCTCAGGGATGCT
TGFB1	TACCTGAACCCGTGTTGCTCTC	GTTGCTGAGGTATCGCCAGGAA
CDK1	GGAAACCAGGAAGCCTAGCATC	GGATGATTCAGTGCCATTTTGCC
CDK4	CCATCAGCACAGTTCGTGAGGT	TCAGTTCGGGATGTGGCACAGA
CDK8	GCTGATAGGAAGGTGTGGCTTC	CCGAGGTAACTGAACTGGCTTC
RB1	CAGAAGGTCTGCCAACACCAAC	TTGAGCACACGGTCGCTGTTAC
CDKN1A	AGGTGGACCTGGAGACTCTCAG	TCCTCTTGGAGAAGATCAGCCG
CDKN2A	CTCGTGCTGATGCTACTGAGGA	GGTCGGCGCAGTTGGGCTCC
WEE1	GATGTGCGACAGACTCCTCAAG	CTGGCTT CCATGTCTT CACCAC
ATM	TGTTCCAGGACACGAAGGGAGA	CAGGGTTCTCAGCACTATGGGA
NFKB1	GCAGCACTACTT CTTGACCACC	TCTGCTCCTGAGCATTGACGTC
CASP3	GGAAGCGAATCAATGGACTCTGG	GCATCGACATCTGTACCAGACC
CASP8	AGAAGAGGGTCATCCTGGGAGA	T CAGGACTTCCTT CAAGGCTGC
CASP9	GTTTGAGGACCTTCGACCAGCT	CAACGTACCAGGAGCCACTCTT
HSP90AA1	TCTGCCTCTGGTGATGAGATGG	CGTTCCACAAAGGCTGAGTTAGC
GAPDH	GTCTCCTCTGACTTCAACAGCG	ACCACCCTGTTGCTGTAGCCAA

## Data Availability

The datasets utilized and/or analyzed during the present study are available from the corresponding author upon reasonable request.
